# A real-time survey on the psychological impact of mild lockdown for COVID-19 in the Japanese population

**DOI:** 10.1038/s41597-020-00714-9

**Published:** 2020-10-29

**Authors:** Nagisa Sugaya, Tetsuya Yamamoto, Naho Suzuki, Chigusa Uchiumi

**Affiliations:** 1 Unit of Public Health and Preventive Medicine, School of Medicine, Yokohama City University, Yokohama, Japan; 2 Graduate School of Technology, Industrial and Social Sciences, Tokushima University, Tokushima, Japan; 3 Faculty of Integrated Arts and Sciences, Tokushima University, Tokushima, Japan

**Keywords:** Epidemiology, Risk factors

## Abstract

To deter the spread of the coronavirus disease 2019 (COVID-19), many countries have imposed a lockdown with restrictions. On 7 April 2020, the Japanese government declared a state of emergency over the COVID-19 outbreak. Japan was in “mild lockdown” which was not enforceable and non-punitive with the declaration. We conducted an online survey to investigate factors associated with psychological distress in the “mild lockdown” under a declared state of emergency for COVID-19. We collected data on 11,333 inhabitants (52.4% women, 46.3 ± 14.6 years) living in the seven prefectures where the declaration was first applied. The investigation dates of this study, 11 and 12 May 2020, were in the final phase of the state of emergency. The survey was conducted in real-time to minimize participants’ recall bias. In addition to psychological inventories often used worldwide, the questionnaires used in this survey included lifestyle and stress management items related to COVID-19 and various socio-demographic items including occupation (e.g. healthcare worker) or income.

## Abstract (summary)

**Table:** 

Measurement(s)	Stress • socio-demographic data • psychological distress • loneliness measurement • social network analysis • Lifestyle
Technology Type(s)	survey method
Factor Type(s)	sex • age
Sample Characteristic - Organism	Homo sapiens
Sample Characteristic - Environment	mild lockdown
Sample Characteristic - Location	Japan

Machine-accessible metadata file describing the reported data: 10.6084/m9.figshare.12996998

## Background & Summary

The coronavirus disease 2019 (COVID-19) continues to spread worldwide^1^. To deter the spread of COVID-19, many countries have imposed a lockdown with restrictions on outings, service closure, etc. The lockdown in most of these countries has compelling force with penalties for violations. The lockdown can be expected to deter the spread of the infection, which would become destructive; not only economic damage (e.g. Gross Domestic Product [GDP] loss) but also psychological distress^2–7^.

**Fig. 1:**
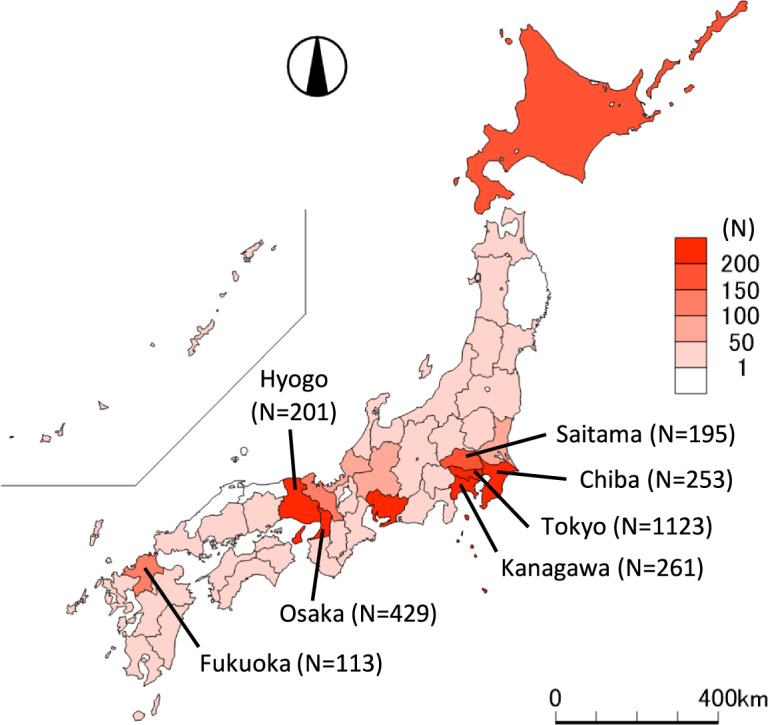
Cumulative number of PCR test positives on 7 April 2020 in seven prefectures where the emergency declaration was first applied^32^.

Japan was in “mild lockdown,” which was not enforceable and non-punitive, with the declaration of a state of emergency, and the impact attracted attention^8^. On 7 April 2020, the Japanese government declared a state of emergency over the COVID-19 outbreak for the seven prefectures (Tokyo, Kanagawa, Osaka, Saitama, Chiba, Hyogo, and Fukuoka; Fig. 1)^9^. The state of emergency expanded nationwide on 16 April 2020, and was lifted in a phased manner on 14 May 2020. While many countries were in the lockdown with penalties for violations, Japanese policy for COVID-19 was distinguished as the government “requested” to refrain from going out except for emergencies and to temporarily close certain businesses without penalties for violations. This lockdown significantly transformed activity in Japan. For example, the number of monthly train users in April 2020 prominently decreased by 45.5% compared with the same month last year^10^. The mild lockdown in Japan, which was not enforceable and non-punitive, had a diverse range of influences on people’s lives like other countries, including changes in domestic circumstances due to teleworking or school closure and economic damage due to decreased income or job loss.

Previous studies have already investigated the association between lockdown and psychological distress. It is reported that lockdown is potentially associated with severe psychological symptoms, including depression or anxiety^3–7^ and possibly with decreased psychological happiness^2^. Additionally, loneliness and social isolation, which are strongly associated with anxiety, depression, self-harm, and suicide attempts throughout one’s life^11,12^, may be increased in lockdown^13,14^. Previous surveys conducted in countries in enforceable lockdowns. However, no studies have investigated the effects of unenforceable mild lockdown on psychological distress. There is an urgent need to investigate the association between lifestyle changes related to mild lockdown, psychological status, and socio-demographic profile in the mild lockdown that affects people’s lives despite its non-punitive nature. In particular, there is inadequate investigation of the stressors and stress coping during the lockdown. Such analysis can contribute to effective provisions of mental health services in a future pandemic.

Thus, to investigate relative factors to psychological distress in mild lockdown we conducted an online survey of inhabitants living in the seven prefectures where the emergency declaration was first applied. We collected data between 11 and 12 May 2020, the period in the final phase of the state of emergency.

## Methods

### Participants and data collection

A total of 11,333 individuals participated in our study (52.4% women, mean age = 46.3 ± 14.6 years, range = 18–89 years). The survey was conducted online between 11 May and 12 May 2020. The survey was designed to assess the psychological impact of the mild lockdown on participants for approximately one month or from the start of “mild lockdown.” The exclusion criteria were as follows: (a) aged <18 years, (b) high school students, and (c) living outside the seven prefectures. To sensitively detect the impact of the mild lockdown, participants were recruited only in the seven prefectures where the emergency declaration was first applied (Tokyo, Kanagawa, Osaka, Saitama, Chiba, Hyogo, and Fukuoka). These prefectures were assumed to be susceptible to mild lockdown due to their large populations and the large number of cases reported in these areas. The number of people collected in each prefecture was determined according to the ratio of the number of people living in Tokyo (n = 2,783, 24.6%), Kanagawa (n = 1,863, 16.4%), Osaka (n = 1,794; 15.8%), Saitama (n = 1,484; 13.1%), Chiba (n = 1,263; 11.1%), Hyogo (n = 1,119; 9.9%), and Fukuoka (n = 1,027; 9.1%).

Through Macromill.inc. (Tokyo, Japan), approximately 80,000 people were recruited by email, and data were collected on an online platform. Participants completed the online survey on the second day after receiving a link to the online survey. All participants voluntarily responded to the survey anonymously and provided informed consent online before the survey. Participants received a clear explanation of the survey procedure and could interrupt or terminate the survey at any time without explaining the reason.

This study was approved by the Research Ethics Committee at the Graduate School of Social and Industrial Science and Technology, Tokushima University (acceptance number 212), and was performed in accordance with the ethical standards of the 1964 Declaration of Helsinki and its later amendments.

### Measurements

#### Socio-demographic data

Participants’ socio-demographic information was collected, including age, sex, employment status, marital status, and annual household income. To compare the impact on the group assumed to be vulnerable to the effects of lockdown in previous studies^2,4,13,14^, information was collected on whether the individual or a family member was a healthcare worker, whether the individual was currently being treated for a mental problem or severe physical disease, and whether the individual had a history of treatment for a mental problem or severe physical disease.

#### Psychological distress

Psychological distress was measured using the Japanese version of the Kessler Psychological Distress Scale-6 (K6)^15^ non-specific psychological stress scale, a six-item screening instrument measuring distress over the past 30 days. Each question was rated on a scale of 0 (none of the time) to 4 (all of the time); total scores ranged from 0 to 24. Owing to its brevity and high accuracy, the K6 is considered an ideal scale for screening for mental disorders in population-based health surveys^15–17^. In addition, because the duration of symptoms examined by this scale (the past 30 days) corresponds to the period between the start of mild lockdown and the implementation of the survey (approximately 1 month), we assumed that the scale would sensitively reflect the influence of psychological distress caused by the mild lockdown. We adopted a threshold of five points commonly used to screen for mild-to-moderate mood/anxiety disorders^18^. K6 scores ranging from 5 to 12 were defined as mild-to-moderate psychological distress (MMPD). This threshold is the optimal lower threshold cutoff point for screening for moderate psychological distress^18^. MMPD is considered because of the associated risk of progression to more severe disability as well as current distress and disability^19^. A threshold score of 13 is a criterion traditionally used in previous studies^16,20^. A score of ≥13 was defined as serious psychological distress (SPD). Additionally, a score of ≤4 was defined as no or low psychological distress.

We also used the Japanese version of the Patient Health Questionnaire-9 (PHQ-9)^21^ to collect basic information on the mental health of participants; the PHQ-9 consists of nine questions. Depressive symptoms during the past four weeks were reported by the participants, with a score of 0 (not at all) to 3 (nearly every day)^22^. We defined a score of ≥10, as recommended by previous studies^21^, as a cutoff point, meaning that a person is more likely to have major depression. The PHQ-9 has been widely used internationally as a screening scale for depression^23^ and is highly reliable and valid^21^.

#### Loneliness and social networks

We measured loneliness since the declaration of the state of emergency on 7 April 2020 using the Japanese version of the UCLA loneliness scale version 3 (UCLA-LS3)^24^. The UCLA-LS3 consists of 10 items, each rated from 1 (never) to 4 (always)^25^. The scores range from 10 to 40, with higher scores indicating higher levels of loneliness. The UCLA-LS3 is highly reliable and valid^24^, and is an internationally used scale for measuring loneliness^26–28^.

We also measured social networks since the declaration of the state of emergency using the Japanese version of the abbreviated Lubben Social Network Scale (LSNS-6)^29^. The LSNS-6 consists of three items related to the family network, three items related to the friendship network, and the number of people in the network is calculated using a six-point scale (0 = none to 5 = nine or more) for each item^30^. The total score ranges from 0 to 30 points, with higher scores indicating a larger social network and <12 points indicating social isolation. The LSNS-6 is highly reliable and valid^29^ and has been used in many countries^31–33^.

#### Lifestyle, stress management, and stressors related to mild lockdown

With extensive reference to the literature on the COVID-19 pandemic^2,4,6,14,34^, we developed eight lifestyle and stress management items and seven stressors assumed to be associated with mild lockdown (Table 1). Item 7 (Optimism) was included in the eight lifestyle and stress management items because optimism acts to reduce depression after experiencing a stressful event^35^. We asked participants to rate the frequency of implementation and experience of these items from the start of the mild lockdown to the time of the survey on a scale of 1 (not at all) to 7 (extremely).

**Table 1:** Items about lifestyle, stress management, and stressors related to mild lockdown.

1.	I exercised for my health (whether indoors or outdoors).
2.	I took meals considering the nutrition balance.
3.	I kept regular awakening time and bedtime approximately.
4.	I engaged in activities such as hobbies with absorbing interest.
5.	I interacted with my family or friends on a face-to-face basis (except work or class).
6.	I interacted with my family or friends online using chat or video calling (except work or class).
7.	I spontaneously refrained from going out or took preventive behaviors (e.g. wearing a mask) to prevent coronavirus disease 2019 infection to my family or other people.
8.	I thought about the future positively.
9.	The family budget has tightened.
10.	A personal relationship with a close person such as family or friends got worse.
11.	I have become easily annoyed or irate due to life-change.
12.	I felt nervous or anxious when I watched news about coronavirus disease 2019.
13.	I could not sleep because I worried about getting coronavirus disease 2019.
14.	My daily life was interrupted due to the shortage of materials relating to prevention for coronavirus disease 2019 infection (e.g. mask or thermometer) or other daily supplies.
15.	My work or schoolwork was interrupted due to life-change.

Items 1–8: Lifestyle and stress management during mild lockdown.

Items 9–15: Stressors related to mild lockdown.

The above methods are elaborated versions of descriptions provided in our related work (ref. ^8^)

## Data Records

Data records are available in XLSX format from the Open Science Framework (OSF) platform together with files of the questionnaires^36^. The datasets were anonymized to remove any personal information. Abbreviation guides for variable names are also included in each XLSX file.

## Technical Validation

### Characteristics of the data

A strength of this data is to be able to evaluate the effect of mild lockdown in real-time by minimizing recall bias. Moreover, the investigation dates of this study, 11 and 12 May 2020, were also in the final phase of the state of emergency when the effect of changes in life due to mild lockdown may be amplified. Additionally, psychological questionnaires applied to this survey have been often used worldwide in psychological or psychiatric researches. Thus, our data is comparable with the results in other countries with enforceable lockdowns for COVID-19.

### Descriptive results

In our dataset, although 1,707 participants (15.1%) did not provide any data regarding annual household income, there were no missing data in other variables.

The socio-demographic characteristics and sex differences using the χ^2^ test are shown in Table 2. There were significant sex differences in all socio-demographic variables except two variables: “the presence of health care worker in participants’ family” and “current treatment of psychological problems.” The “Unknown” of annual household income in Table 2 includes the missing values (N = 1707).

**Table 2:** Socio-demographic characteristics and sex difference.

	N (%)						Sex difference		
	Total		Male		Female		*χ*^2^	*p*	*φ*
Overall	11333	(100)	5391	(100)	5942	(100)			
Age							1071.35	<0.001	0.307
*18-19*	143	(1.3)	46	(0.9)	97	(1.6)		*	
*20-39*	3745	(33.0)	1031	(19.1)	2714	(45.7)		*	
*40*–*64*	6024	(53.2)	3295	(61.1)	2729	(45.9)		*	
≥*65*	1421	(12.5)	1019	(18.9)	402	(6.8)		*	
Occupation							2115.58	<0.001	0.432
*Employed*	7685	(67.8)	4235	(78.6)	3450	(58.1)		*	
*Homemaker*	1806	(15.9)	25	(0.5)	1781	(30.0)		*	
*Student*	407	(3.6)	122	(2.3)	285	(4.8)		*	
*Unemployed*	1068	(9.4)	808	(15.0)	260	(4.4)		*	
*Other*	367	(3.2)	201	(3.7)	166	(2.8)		*	
Healthcare worker (Yes)^†^									
*Self*	661	(5.8)	200	(3.7)	461	(7.8)	84.35	<0.001	0.086
*Family*	991	(8.7)	455	(8.4)	536	(9.0)	1.19	0.287	0.010
Marital status (Married)^†^	7043	(62.1)	3492	(64.8)	3551	(59.8)	30.20	<0.001	0.052
Annual household income (JPY)							426.96	<0.001	0.194
<*2.0 million*	633	(5.6)	308	(5.7)	325	(5.5)			
*2.0*–*3.9 million*	1990	(17.6)	947	(17.6)	1043	(17.6)			
*4.0*–*5.9 million*	2214	(19.5)	1150	(21.3)	1064	(17.9)		*	
*6.0*–*7.9 million*	1495	(13.2)	818	(15.2)	677	(11.4)		*	
≥*8.0 million*	2130	(18.8)	1247	(23.1)	883	(14.9)		*	
*Unknown*	2871	(25.3)	921	(17.1)	1950	(32.8)		*	
Treatment of severe physical diseases (Yes)^†^									
*Current*	482	(4.3)	344	(6.4)	138	(2.3)	114.33	<0.001	0.100
*Previous*	851	(7.5)	563	(10.4)	288	(4.8)	127.47	<0.001	0.106
Treatment of mental problems (Yes)^†^									
*Current*	641	(5.7)	317	(5.9)	324	(5.5)	0.97	0.329	0.009
*Previous*	1366	(12.1)	582	(10.8)	784	(13.2)	15.34	<0.001	0.037

^†^Differences between total number and the numbers indicated in this table are the numbers of “No” or “Not married” because there are no missing data regarding these questions.

*Significant sex difference found by residual analysis (adjusted residual > 1.96).

The size criteria for *ϕ* are: 0.100 = small, 0.300 = medium, 0.600 = large.

Online-only Table 1 displays the descriptive results of psycho-social indexes and items specific to mild lockdown and sex differences in these variables using the *t*-test. Sex differences were significant in these variables except “healthy sleep habits,” one of the items specific to mild lockdown. In total, 4,146 participants (36.6%) had MMPD (K6 score 5–12) and 1,303 (11.5%) had SPD (K6 score ≥13). In previously published data in 2019 concerning K6 in the Japanese population from the Ministry of Health, Labour and Welfare (217,179 households), 26.9% of participants had SPD or MMPD (i.e., K6 score ≥ 5)^37^. Additionally, the estimated prevalence of depression (PHQ-9 score ≥10) was 2,034 (17.9%). In a previous survey of the general Japanese population conducted in 2013 (N = 3753), 7.9% of participants reported a PHQ-9 score of ≥10^38^.

Online-only Table 2 displays the Pearson’s correlation coefficients between psycho-social indexes and items specific to mild lockdown. There were significant correlations between these variables except between the K6 score and “online interaction with familiar people” or “preventive behaviors of COVID-19” and between the LSNS-6 score and “difficulties owing to the lack of daily necessities.” There were moderate correlations between the K6 score and “frustration” or “COVID-19-related sleeplessness” scores, between the PHQ-9 score and “frustration” score, and between the UCLA-LS3 score and “optimism” score.

Therefore, sex differences in many socio-demographic variables and psychological and lifestyle items in our data were statistically significant. Moreover, psychological distress indices significantly correlated with several items relating to COVID-19. In the hypothesis testing using our dataset or the comparison with other datasets, our results and particularly sex differences in age should be considered.

## Online-only Tables

**Online-only Table 1:** Descriptive results of psycho-social indexes and items specific to mild lockdown and sex difference.

	Mean score (SD)						Sex difference			
	Total		Male		Female		Difference (95%CI)		*p*	*Cohen’s d*
Psychological distress										
*K6*	5.58	(5.43)	4.89	(5.19)	6.21	(5.57)	−1.32	(−1.52, −1.12)	<0.001	0.246
*PHQ-9*	4.90	(5.53)	4.37	(5.39)	5.39	(5.62)	−1.02	(−1.22, −0.82)	<0.001	0.185
Loneliness and social network										
*UCLA-LS3*	23.46	(5.70)	23.97	(5.52)	22.99	(5.82)	0.98	(0.77, 1.19)	<0.001	0.172
*LSNS-6*	10.56	(6.17)	9.65	(6.31)	11.38	(5.92)	−1.73	(−1.96, −1.51)	<0.001	0.283
Lifestyle and stress management during mild lockdown										
*1. Exercise*	4.17	(1.81)	4.11	(1.83)	4.22	(1.80)	−0.10	(−0.17, −0.04)	0.002	0.058
*2. Healthy eating habits*	4.34	(1.56)	4.12	(1.56)	4.53	(1.52)	−0.41	(−0.47, −0.36)	<0.001	0.268
*3. Healthy sleep habits*	4.63	(1.79)	4.63	(1.76)	4.62	(1.82)	0.005	(−0.06, 0.07)	0.890	0.003
*4. Activity*	4.02	(1.67)	3.99	(1.63)	4.06	(1.71)	−0.07	(−0.13, −0.01)	0.024	0.042
*5. Offline interaction with familiar people*	3.53	(1.88)	3.57	(1.79)	3.50	(1.95)	0.07	(0.00, 0.14)	0.044	0.038
*6. Online interaction with familiar people*	3.27	(2.00)	2.95	(1.86)	3.56	(2.09)	−0.60	(−0.67, −0.53)	<0.001	0.305
*7. Preventive behaviors of COVID-19*	5.58	(1.67)	5.16	(1.76)	5.96	(1.47)	−0.81	(−0.87, −0.75)	<0.001	0.496
*8. Optimism*	4.06	(1.57)	3.98	(1.55)	4.12	(1.59)	−0.14	(−0.20, −0.08)	<0.001	0.090
Stressors related to mild lockdown										
*9. Deterioration of household economy*	3.80	(1.83)	3.69	(1.79)	3.89	(1.86)	−0.20	(−0.27, −0.13)	<0.001	0.111
*10. Deterioration of relationship with familiar people*	2.38	(1.54)	2.47	(1.53)	2.30	(1.56)	0.17	(0.11, 0.22)	<0.001	0.108
*11. Frustration*	3.31	(1.75)	3.02	(1.67)	3.58	(1.79)	−0.56	(−0.63, −0.50)	<0.001	0.326
*12. COVID-19-related anxiety*	4.04	(1.70)	3.63	(1.67)	4.41	(1.64)	−0.79	(−0.85, −0.73)	<0.001	0.476
*13. COVID-19-related sleeplessness*	2.44	(1.54)	2.36	(1.45)	2.52	(1.61)	−0.16	(−0.21, −0.10)	<0.001	0.102
*14. Difficulties owing to the lack of daily necessities*	3.63	(1.85)	3.32	(1.77)	3.91	(1.88)	−0.59	(−0.66, −0.53)	<0.001	0.326
*15. Difficulties in work or schoolwork*	3.82	(2.05)	3.55	(1.94)	4.07	(2.12)	−0.52	(−0.59, −0.44)	<0.001	0.256

COVID-19, coronavirus disease 2019; K6, Kessler Psychological Distress Scale-6; PHQ-9, Patient Health Questionnaire-9; UCLA-LS3, UCLA Loneliness Scale (version 3); LSNS-6, Lubben Social Network Scale (abbreviated version); CI, confidence interval

The numbers 1–15 in this table are the item numbers of the questionnaire about lifestyle, stress management, and stressors related to mild lockdown (Table 1).

The effect size criteria for Cohen’s d are: 0.200 = small, 0.500 = medium, 0.800 = large.

**Online-only Table 2:** Correlation between psycho-social indexes and items specific to mild lockdown.

		1. Exercise	2. Healthy eating habits	3. Healthy sleep habits	4. Activity	5. Offline interaction with familiar people	6. Online interaction with familiar people	7. Preventive behaviors of COVID-19	8. Optimism
K6	*r*	−0.070	−0.092	−0.209	−0.108	−0.075	−0.018	−0.013	−0.247
	*p*	<0.001	<0.001	<0.001	<0.001	<0.001	0.057	0.170	<0.001
PHQ-9	*r*	−0.121	−0.153	−0.244	−0.163	−0.085	−0.049	−0.040	−0.270
	*p*	<0.001	<0.001	<0.001	<0.001	<0.001	<0.001	<0.001	<0.001
UCLA-LS3	*r*	−0.238	−0.295	−0.253	−0.257	−0.243	−0.302	−0.217	−0.425
	*p*	<0.001	<0.001	<0.001	<0.001	<0.001	<0.001	<0.001	<0.001
LSNS-6	*r*	0.259	0.269	0.187	0.249	0.269	0.354	0.261	0.354
	*p*	<0.001	<0.001	<0.001	<0.001	<0.001	<0.001	< 0.001	<0.001
		**9. Deterioration of household economy**	**10. Deterioration of relationship with familiar people**	**11. Frustration**	**12. COVID-19-related anxiety**	**13. COVID-19-related sleeplessness**	**14. Difficulties owing to the lack of daily necessities**	**15. Difficulties in work or schoolwork**	
K6	*r*	0.272	0.370	0.492	0.370	0.400	0.301	0.262	
	*p*	<0.001	<0.001	<0.001	<0.001	<0.001	<0.001	<0.001	
PHQ-9	*r*	0.246	0.347	0.430	0.267	0.335	0.265	0.231	
	*p*	<0.001	<0.001	<0.001	<0.001	<0.001	<0.001	<0.001	
UCLA-LS3	*r*	0.153	0.297	0.261	0.091	0.189	0.129	0.084	
	*p*	<0.001	<0.001	<0.001	<0.001	<0.001	<0.001	<0.001	
LSNS-6	*r*	−0.035	−0.148	−0.074	0.046	−0.059	−0.0004	0.063	
	*p*	<0.001	<0.001	<0.001	<0.001	< 0.001	0.967	<0.001	

COVID-19, coronavirus disease 2019; K6, Kessler Psychological Distress Scale-6; PHQ-9, Patient Health Questionnaire-9; UCLA-LS3, UCLA Loneliness Scale (version 3); LSNS-6, Lubben Social Network Scale (abbreviated version)

Pearson’s correlation coefficient.

The numbers 1–15 in this table are the item numbers of the questionnaire about lifestyle, stress management, and stressors related to mild lockdown (Table 1).
